# The natural caesarean: a woman-centred technique

**DOI:** 10.1111/j.1471-0528.2008.01777.x

**Published:** 2008-07

**Authors:** J Smith, F Plaat, NM Fisk

**Affiliations:** a Division of Maternity, Directorate of Women's and Children's Services, Queen Charlotte's and Chelsea HospitalLondon, UK; b Department of Anaesthesia, Queen Charlotte's and Chelsea HospitalLondon, UK; c Division of Surgery, Oncology, Reproductive Biology and Anaesthetics, Institute of Reproductive and Developmental Biology, Imperial College LondonHammersmith Campus, London, UK

**Keywords:** Bonding, caesarean section, natural childbirth, physiological resuscitation, skin-to-skin contact

## Abstract

Although much effort has gone into promoting early skin-to-skin contact and parental involvement at vaginal birth, caesarean birth remains entrenched in surgical and resuscitative rituals, which delay parental contact, impair maternal satisfaction and reduce breastfeeding. We describe a ‘natural’ approach that mimics the situation at vaginal birth by allowing (i) the parents to watch the birth of their child as active participants (ii) slow delivery with physiological autoresuscitation and (iii) the baby to be transferred directly onto the mother's chest for early skin-to-skin. Studies are required into methods of reforming caesarean section, the most common operation worldwide.

*Please cite this paper as*: Smith J, Plaat F, Fisk N. The natural caesarean: a woman-centred technique. BJOG 2008;115:1037–1042.

## Introduction

The management of vaginal birth has changed hugely in the 15 years since woman-centred maternity services were recommended in the Department of Health's Changing Childbirth report.^1^ Caesarean rates doubled during the same period; yet, abdominal delivery has changed little, apart from occasional background music and a safety-led shift from general to regional anaesthesia.

If promoting what is natural underpins the modern management of vaginal birth, the need for speed and resuscitation remain the principles governing techniques for caesarean birth today. Surgical rapidity, however, is unnecessary in the absence of fetal compromise, and a throwback to the days when general anaesthesia was the norm, with short induction-delivery intervals advocated to reduce fetal anaesthetic exposure and the subsequent need for resuscitation. Although paediatricians are no longer required at straightforward caesareans under regional block,^2^ the baby is usually taken to a Resuscitaire, examined, cleaned, tagged, weighed and swaddled before being introduced to the parents, often a good 10 minutes after birth. Early skin-to-skin contact and initiation of breastfeeding within 30 minutes as recommended by the WHO/UNICEF Baby Friendly Initiative^3^,^4^ is almost nonexistent.^5^

Increasing evidence shows that women undergoing caesareans have a less satisfactory childbirth experience than those delivering vaginally and are more prone to postnatal depression, bonding difficulties and unsuccessful breastfeeding.^6^,^7^ To improve the experience of women having uncomplicated caesareans, we have modified obstetric, midwifery and anaesthetic practice over the past 6 years to emulate as closely as practicable the woman-centred aspects of ‘natural’ vaginal birth.

## Technique

We describe a technique for straightforward elective caesareans in healthy women with non-compromised singleton fetuses at term. It can be adapted for nonurgent emergency procedures but is not suitable for preterm or breech presentations.

### Preparation

Antenatally, we use video clips to demonstrate what happens at a ‘natural’ caesarean. When possible, the woman (and her partner) meet the midwife and obstetrician preoperatively and are shown the operating theatre to render the environment less intimidating. The couples are encouraged to bring their own music, and the woman can wear her own clothing if she wishes.

In theatre, the pulse oximeter is positioned on the mother's foot to keep her hands free, and the electrocardiogram (ECG) leads away from her anterior chest wall where the baby will be placed. The anaesthetic block aims to permit pain-free surgery without requiring supplementation (which may obtund the woman's responses). It should not affect the upper limbs needed to hold her baby nor cause haemodynamic instability with its potential for light-headedness, nausea or vomiting. The intravenous line is placed in the nondominant arm as per usual practice. We use a combined spinal-epidural needle-through-needle technique with 7.5–10 mg bupivacaine intrathecally^8^ and a prophylactic infusion of the vasopressor phenylephrine. Once the block is sited, one of the woman's arms is freed from her clothing to facilitate skin-to-skin contact. Cardiotocography is continued until skin preparation to confirm fetal wellbeing.

### Delivery: walking the baby out

**Figure 1:**
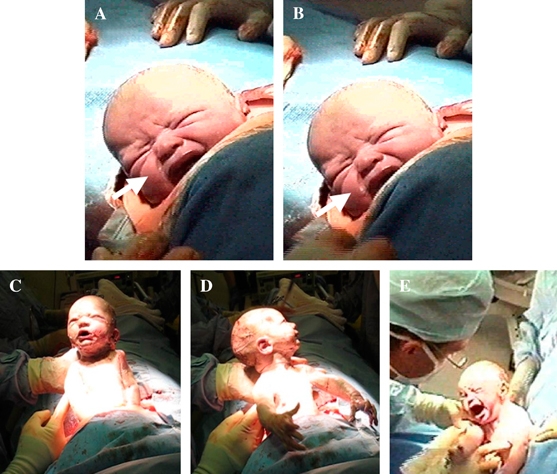
Autoresuscitation. After delivery of the head, the baby establishes respiration while still attached to the placental circulation. Pausing with the head in this position allows external compression from the uterus and maternal soft tissues to expel lung liquid (arrows A and B—time lapse) as happens at vaginal delivery. Note that neither the surgeon nor the assistant is touching the baby. The baby's trunk is then eased out by a combination of uterine contractions and gentle assistance from the accoucheur to ensure it facies the watching parents (C). The baby often unleashes his/her own arms from the uterus with a vigorous extension reflex (D), and his/her wellbeing is monitored by observing crying and facial reactions (E). Representative photographs from different deliveries (with permission).

Surgery starts with the screen up, and sterile routines observed as usual. After uterine incision, the drape is lowered and the head of the table raised to enable the mother to watch the birth. As the fetal head enters the abdominal incision, the operative field is cleaned of blood and the partner is invited to stand to observe the birth. The principle for the surgeon is then hands-off, as the baby autoresuscitates: breathing air through the exteriorised mouth and nose, while its trunk still *in utero* remains attached to the placental circulation. This delay of a few minutes allows pressure from the uterus and maternal soft tissues to expel lung liquid (Figure 1), mimicking what happens at vaginal delivery. Once crying, the baby's shoulders are eased out, and the baby then frequently delivers his/her own arms with an expansive gesture. Concurrently, the baby's torso tamponades the uterine incision, minimising bleeding (Figure 1).

**Figure 2:**
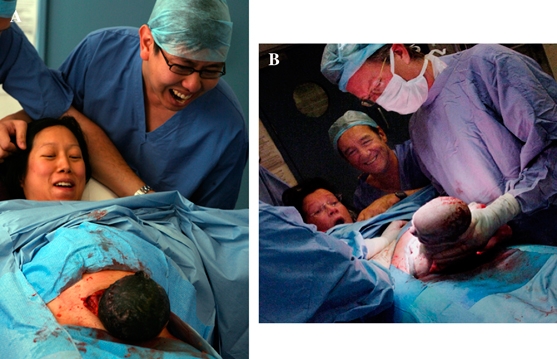
Parental participation. Dropping the drape and tilting the head of the bed upwards allows the parents to establish eye contact and learn of the baby's sex as he/she emerges. The father may stand if he wishes. (A) and (B) show representative photographs from different deliveries (with permission).

The baby is next left supported for up to a minute, allowing the mother to observe her child. The half-delivered fetus frequently cries but if not, the obstetrician observes its breathing, colour, tone and movement to indicate wellbeing. The rest of the delivery is achieved through a combination of passive expulsion by the contracting uterus and active assistance: the baby wriggles out while its head and torso are supported by the obstetrician. This enables the mother to watch the birth and ascertain the sex of her baby at the same time as the delivery team, replicating the situation at vaginal birth (Figure 2).

### Early skin-to-skin contact

**Figure 3:**
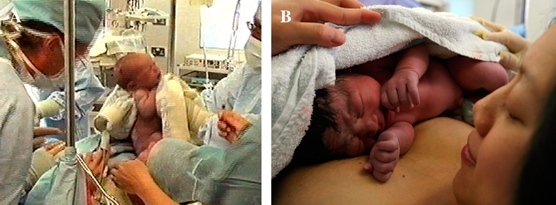
Early skin-to-skin contact. The baby is handed by the surgeon (left) first to the midwife (right) waiting alongside the mother's head (A), then directly to mother. Skin-to-skin contact is established within a minute of delivery. The screen is then restored while surgical closure is completed, and the baby kept warm with towels and bubble wrap (B). Representative photographs from different deliveries (with permission).

Once the baby is finally ‘born’ and wellbeing again confirmed, the cord is clamped and cut in view of the parents. The anaesthetist/anaesthetic assistant clears the mother's clothing from her chest, and the midwife positions him/herself at the top of the bed beside the mother's head. Still scrubbed, the midwife receives the baby directly from the surgeon to prevent contamination (Figure 3). The woman should be warned not to reach out for her baby, as this risks touching the obstetrician. The baby is laid prone between the mother's breasts, dried with a warmed towel and kept warm with fresh towels and bubble wrap.

After a plastic clamp is applied, the partner can cut the remaining cord if he wishes. Labelling and vitamin K administration are accomplished with the baby on the mother's chest. The baby is positioned so that he/she can begin to suckle. The midwife remains near the head end to monitor the baby and reassure the parents. The baby is only weighed when surgery is finished, and given to the partner while the mother is transferred to her bed. Skin-to-skin contact is then re-established with the baby in the same position.

## Discussion

Caesarean section remains entrenched in hospital routines, seemingly immune to the tide of customer-focused changes that has swept maternity services and labour ward care. The ‘natural’ caesarean technique we describe has evolved as a series of measures to mimic the situation at vaginal birth, where birth attendants encourage early skin-to-skin contact, facilitate physiological resuscitation, but most of all, engage the parents as active participants in the birth of their child.

Randomised trials demonstrate that early skin-to-skin contact increases the rate and duration of breastfeeding, reduces infant crying and improves maternal affection. Although recommended by both the Royal College of Nursing and National Institute for Clinical Excellence,^2^,^9^ this has hitherto proved refractory to implement at caesarean section against a background of ritualised obstetric and midwifery routines.^5^ We describe how immediate skin-to-skin contact can be established at straightforward abdominal delivery without compromising operative sterility.

Respiratory complications like transient tachypnoea of the newborn are more common after elective caesarean than vaginal delivery,^10^–^12^ in which retained lung liquid is implicated, as is the lack of catecholamine and cortisol surge associated with vaginal birth.^13^ Pausing the delivery of the baby, as shown in Figure 3, to allow physiological expulsion of lung liquid like at vaginal delivery may facilitate respiratory adaptation.

We report the technique at this stage in the absence of quantitative outcome data in response to frequent requests from obstetricians and women, and considerable media interest. In qualitative terms, the natural caesarean has been positively received by the couples involved (Supplementary material S1), with no adverse comment in more than 100 procedures. One fear that women expressed preoperatively was the possibility that they would see inside their own abdomen. We explain that the baby's head ‘blocks the hole’ and once baby is delivered, the screen goes back up. In reality, maternal position precludes this anyway. Formal audit of maternal and fetal outcomes is now indicated, with a view to eventual randomisation. We offer the following observations on introducing this package of measures.

Fetal safety is paramount, and we immediately resort to routine management if the baby is unexpectedly born in poor condition. The uterine incision-to-delivery interval is prolonged compared with routine practice, but usually still within the 3 minutes formerly recommended for optimal neonatal condition.^14^ However, during this period with the head out but the trunk still inside, not only is the crying baby establishing a resting lung volume but also the placental circulation remains intact. Experience with the partially exteriorised fetus during EXIT (Ex utero Intrapartum Treatment surgery to establish the neonatal airway) procedures suggests that fetal oxygenation can be maintained over much longer intervals, the largest series showing an average cord pH of 7.20 after a median of 17 minutes on ‘placental bypass’.^15^ Because birth is timed when the baby is completely expelled from its mother's womb, natural caesarean babies often achieve a healthy Apgar score before they are actually born.

Thermal care warrants attention. Although neonates undergoing skin-to-skin contact after vaginal birth are no colder than those who do not,^16^ the theatre environment is different from that of a labour room. Air conditioning may increase heat loss through convection drafts, even when ambient temperature is maintained. Following conventional delivery, the baby is routinely placed under the radiant heat of the Resuscitaire before being swaddled. We warm and cover the baby on the mother's chest and maintain theatre temperature ≥25°C.

To establish that the mother's chest is a safe place for the baby while the woman is undergoing surgery, we ensure that the mother wishes to have the baby there and is in a fit state to do so. We request that the partner helps support the baby, and an important practice point is that the midwife remains at the head end after delivery, as the anaesthetist is busy at this juncture. The operating table should be levelled from the preoperative lateral tilt. An unanticipated problem was that the maternal ECG sometimes picks up the baby's heartbeat as he/she lies on the mother's chest, mimicking a potentially alarming maternal tachycardia.

Perhaps, the biggest obstacle to implementation is reluctance of staff to change roles and give up rituals. A multidisciplinary team approach is key. The surgeon cannot remain aloof behind the drape. The anaesthetic team must embrace the presence of the baby at the ‘head-end’, and midwives need to accept that what is good practice at a vaginal birth is also achievable in theatre. An initial midwifery concern that the technique would delay a busy operating list, with no opportunity to weigh, check and dress the baby or complete the paperwork until the end of the operation, was assuaged once the staff had witnessed the family friendly benefits of a ‘natural’ section.

Caesarean section rates are rising worldwide, and indeed, now exceed one-third of deliveries in many developed world centres such as our own. Given the negative effect that caesareans, whether indicated or discretionary, have on maternal satisfaction, bonding and breastfeeding, improving this experience while maintaining safety should be a priority. We describe an evolving approach, suitable for global export. Studies are now indicated on the effects of naturalising this most unnatural form of birth.

## Editor's Commentary

VarnerM

Peripartum care of women and their babies has changed dramatically over the previous half century. While the evolution of prenatal diagnosis and fetal monitoring, safer anaesthesia, and improved surgical techniques have continued to improve and optimise perinatal outcomes, this progressive ‘technicalisation’ of the birth experience has also resulted in many ultimately unnecessary interventions as well as frequent dissatisfaction among women and their families with the institutionalised birth process. Many developed world medical centres have responded with various ‘woman-friendly’, ‘family-friendly’ and ‘baby-friendly’ initiatives, all with the intentions of optimising outcomes and satisfaction for mothers and babies, promoting bonding and breastfeeding, and minimising risks for all involved. These ‘friendly’ initiatives are predicated on healthy mothers and healthy babies, generally assuming the spontaneous onset and normal progression of labour at term and have been carefully monitored to ensure that outcomes are truly optimised (Waldenstrom and Nilsson, *Birth* 1997;24:17–26; Jackson *et al.*, *Am J Public Health* 2003;93:999–1006).

While these initiatives have often been instituted by medical centres reactively rather than proactively, they have performed much to promote what is truly natural in human labour and delivery. Now, however, the worldwide caesarean epidemic is seen by many women as a further threat to their ability to safely and humanely deliver their babies. The group from Queen Charlotte's and Chelsea Hospital describe a surgical technique in this issue that has, by their report, evolved in their institution over the past few years in an attempt to improve the experience of women having ‘uncomplicated’ caesarean births.

The authors are to be commended for their caesarean birth preparation activities. While this can do much to enhance acceptance by women and their partners of caesarean birth, it should not be assumed that caesarean birth is ‘natural’. There is impressive evidence that the risk of serious intraoperative complications increases with the number of previous caesarean births (Silver *et al.*, *Obstet Gynecol* 2006;107:1226–32) and I hope that the option of a trial of labour would also be presented fairly in this setting.

Most importantly, however, no outcomes or safety data are presented to justify widespread utilisation of this technique. While this is acknowledged by the authors, it is critically important that readers understand this. We should demand that these techniques be adequately studied with appropriately powered clinical trials and meaningful outcomes. The history of clinical medicine is littered with examples wherein new approaches were adopted wholesale without adequate evaluation, only later to be found to be of no benefit or to have added increased risks. The reality is that protocols such as described here are evolving in many centres around the world, and it is imperative that they be adequately evaluated.

*BJOG* has a tradition of publishing controversial techniques (Chien, *BJOG* 2006;113:988). Constructive controversy, and the resultant dialogue, is good for everyone and generally accelerates improvement in techniques and outcomes. We look forward to clinical trials that evaluate the changing techniques of caesarean birth.

## Supplementary material

The following supplementary materials are available for this article:

Appendix S1 Patient experiences of “natural caesarean”.

These materials are available as part of the online article from: http://www.blackwell-synergy.com/doi/abs/10.1111/j.1471-0528.2008.01777.x.

(This link will take you to the article abstract).

## References

[1] Department of Health (1993). Changing Childbirth: Report of the Expert Maternity Group.

[2] NICE (2004). Caesarean Section.

[3] WHO/UNICEF (1989). Protecting, Promoting and Supporting Bread-feeding: The Special Role of Maternity Service.

[4] Anderson G, Moore E, Hepworth J, Bergman N (2003). Early skin-to-skin contact for mothers and their healthy newborn infants. Cochrane Database Syst Rev.

[5] Rowe-Murray HJ, Fisher JR (2002). Baby friendly hospital practices: cesarean section is a persistent barrier to early initiation of breastfeeding. Birth.

[6] DiMatteo MR, Morton SC, Lepper HS, Damush TM, Carney MF, Pearson M (1996). Cesarean childbirth and psychosocial outcomes: a meta-analysis. Health Psychol.

[7] Dewey KG, Nommsen-Rivers LA, Heinig MJ, Cohen RJ (2003). Risk factors for suboptimal infant breastfeeding behavior, delayed onset of lactation, and excess neonatal weight loss. Pediatrics.

[8] McNaught AF, Stocks GM (2007). Epidural volume extension and low-dose sequential combined spinal-epidural blockade: two ways to reduce spinal dose requirement for caesarean section. Int J Obstet Anesth.

[9] Bick D (2004). The post-natal health needs of women following caesarean section.

[10] Hansen AK, Wisborg K, Uldbjerg N, Henriksen TB (2007). Elective caesarean section and respiratory morbidity in the term and near-term neonate. Acta Obstet Gynecol Scand.

[11] Hansen AK, Wisborg K, Uldbjerg N, Henriksen TB (2008). Risk of respiratory morbidity in term infants delivered by elective caesarean section: cohort study. BMJ.

[12] Taylor A, Fisk N, Glover V (2000). Mode of delivery and subsequent stress response. Lancet.

[13] Faxelius G, Hagnevik K, Lagercrantz H, Lundell B, Irestedt L (1983). Catecholamine surge and lung function after delivery. Arch Dis Child.

[14] Bader AM, Datta S, Arthur GR, Benvenuti E, Courtney M, Hauch M (1990). Maternal and fetal catecholamines and uterine incision-to-delivery interval during elective cesarean. Obstet Gynecol.

[15] Bouchard S, Johnson MP, Flake AW, Howell LJ, Myers LB, Adzick NS (2002). The EXIT procedure: experience and outcome in 31 cases. J Pediatr Surg.

[16] Carfoot S, Williamson P, Dickson R (2005). A randomised controlled trial in the north of England examining the effects of skin-to-skin care on breast feeding. Midwifery.

