# Supplementary material for: The natural caesarean: a woman-centred technique
Source: BJOG. 2008 Jul;115(8):1037–42. doi: 10.1111/j.1471-0528.2008.01777.x (PMC2613254; doi:10.1111/j.1471-0528.2008.01777.x)
Supplement: Appendix S1 — Patient experiences of “natural caesarean”. [file bjo0115-1037-sd1.doc]

**Supplementary Material S1:**

**Patient experiences of “natural caesarean”**

- “I felt more like a participant in an amazing family experience than a patient having a serious operation.”
- “It was amazing to see and hear him slowly emerge from my tummy; first his head gurgling to clear the fluid from his lungs, then his first breath followed by a healthy cry, one shoulder then another, then him greeting the world with open arms – all whilst his legs were still in my tummy.”
- “Immediately following the moment of (baby’s) birth, he was handed to me and lay on me with skin-to-skin contact. This immediate bonding was so exciting that I was virtually unaware of the surgery that followed.”
- “Pure magic…it felt very calm yet excruciatingly exciting at the same time.”
- “It was the most fascinating connecting feeling to see (baby) first sitting then emerging from my tummy ... slowly tenderly coming up into my arms and onto my chest, skin-on-skin, cosy and serene.”
